# Coordinated Bacteriocin Expression and Competence in *Streptococcus pneumoniae* Contributes to Genetic Adaptation through Neighbor Predation

**DOI:** 10.1371/journal.ppat.1005413

**Published:** 2016-02-03

**Authors:** Wei-Yun Wholey, Travis J. Kochan, David N. Storck, Suzanne Dawid

**Affiliations:** 1 University of Michigan Department of Pediatrics, Ann Arbor, Michigan, United States of America; 2 University of Michigan Department of Microbiology, Ann Arbor, Michigan, United States of America; University of Tubingen, GERMANY

## Abstract

*Streptococcus pneumoniae* (pneumococcus) has remained a persistent cause of invasive and mucosal disease in humans despite the widespread use of antibiotics and vaccines. The resilience of this organism is due to its capacity for adaptation through the uptake and incorporation of new genetic material from the surrounding microbial community. DNA uptake and recombination is controlled by a tightly regulated quorum sensing system that is triggered by the extracellular accumulation of competence stimulating peptide (CSP). In this study, we demonstrate that CSP can stimulate the production of a diverse array of *blp* bacteriocins. This cross stimulation occurs through increased production and secretion of the bacteriocin pheromone, BlpC, and requires a functional competence regulatory system. We show that a highly conserved motif in the promoter of the operon encoding BlpC and its transporter mediates the upregulation by CSP. The accumulation of BlpC following CSP stimulation results in augmented activation of the entire *blp* locus. Using biofilm-grown organisms as a model for competition and genetic exchange on the mucosal surface, we demonstrate that DNA exchange is enhanced by bacteriocin secretion suggesting that co-stimulation of bacteriocins with competence provides an adaptive advantage. The *blp* and *com* regulatory pathways are believed to have diverged and specialized in a remote ancestor of pneumococcus. Despite this, the two systems have maintained a regulatory connection that promotes competition and adaptation by targeting for lysis a wide array of potential competitors while simultaneously providing the means for incorporation of their DNA.

## Author Summary

The bacterial pathogen, *Streptococcus pneumoniae*, is a highly adaptable organism that has managed to survive in the face of significant selective pressure from antibiotics and vaccines due to frequent uptake of new gene sequences. DNA is acquired from the environment by *S*. *pneumoniae* during the competence state when regulatory pathways are triggered that result in the production of proteins required for DNA uptake and integration. In this study, we demonstrate that the secretion of small antimicrobial peptides called bacteriocins is induced during competence. These bacteriocins, which are quite diverse in pneumococcal populations, target neighboring organisms for lysis. The coordinated production of the peptides during competence may allow pneumococcal strains that produce a greater repertoire of bacteriocins a selective advantage by increasing the potential pool of DNA donors.

## Introduction

*Streptococcus pneumoniae* (pneumococcus) is an important human pathogen that resides primarily in the human nasopharynx. The genome of pneumococcus is characterized by significant diversity that is a direct result of frequent genetic exchange with the surrounding microbial community including other pneumococci and related streptococcal species. This diversity results in changes in the capsule locus that allow for host-immune evasion and acquisition of antibiotic resistance genes that allow the organism to persist despite environmental pressures that favor elimination [1,2,3,4]. The microbial community of the nasopharynx represents a competitive environment; in addition to pressure from the host, the outcome of direct and indirect inter-bacterial competition also shapes the makeup of the community. Pneumococcus utilizes a number of strategies to persist in the face of clearance mediated by host and microbiome and serves as an excellent example of how adaptation and active competition can combine to provide the resources for survival.

**Fig 1:**
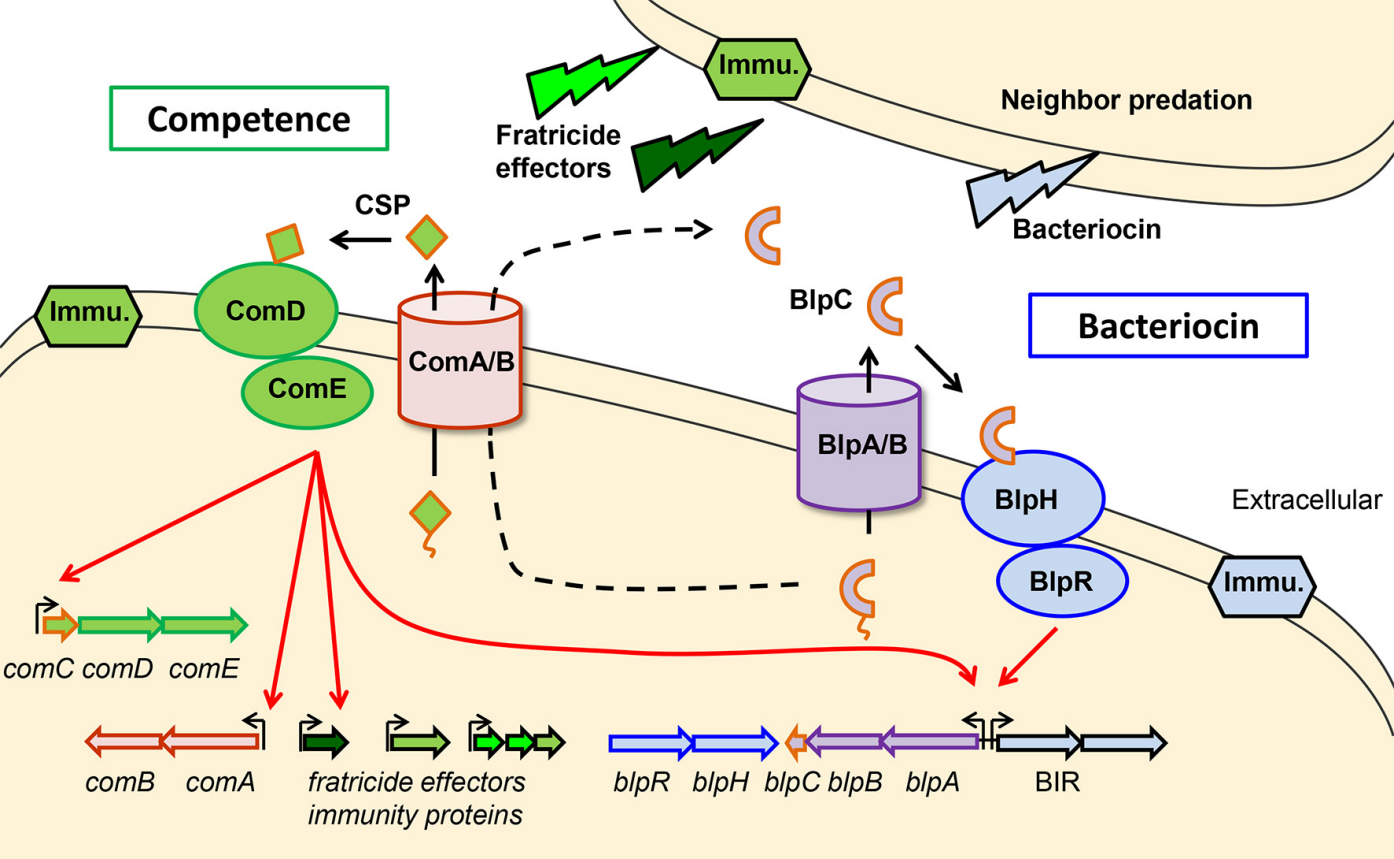
Schematic model of the genetic organization and regulation of the Com and Blp systems. Genes are labeled with colored arrows, colors used match the gene products. Red arrows demonstrate regulatory pathways, black arrows represent secretion pathways. CSP is processed and secreted out of the cell by the ComAB transporter complex. When sufficient local concentrations of CSP accumulate, CSP binding to the receptor ComD activates ComE, a DNA binding protein. ComE then upregulates genes in the competence regulon either directly through binding to promoters or indirectly through induction of an alternative sigma factor. ComE upregulated genes include the genes encoding fratricide effectors and their associated immunity proteins. Blp bacteriocin expression is controlled by the local accumulation of BlpC after secretion and processing by the BlpAB complex. BlpC binds to and activates the two component system, BlpHR. Activated BlpR upregulates the genes in the *blp* locus including genes in the BIR that encode bacteriocins (pneumocins) and immunity proteins. Pneumocins can target neighboring cells that do not produce Blp specific immunity. In this work we provide evidence that activated ComE can upregulate the production of BlpABC through binding to and upregulating the *blpABC* promoter. In addition, we show that ComAB can serve as an alternative transporter for BlpC which may play a particularly important role in the absence of a functional BlpAB.

The competence system in *S*. *pneumoniae* is a highly conserved regulon that controls the production of factors required for DNA uptake and genetic recombination [5,6]. Population studies have shown that interruption of the competence system through phage insertion in critical competence genes results in lineages that show little to no evidence of homologous recombination while lineages with intact competence systems are characterized by extensive evidence of recombination on the chromosome [1,7]. The competence system in pneumococcus is controlled by a quorum sensing system that is regulated by the extracellular accumulation of the competence stimulating peptide pheromone, CSP [8]. The dedicated ABC transporter complex, ComAB, processes and secretes CSP (Fig 1). A 25 amino acid signal sequence preceding the mature CSP sequence is recognized by ComA and cleaved at a double glycine motif while the peptide is transported out of the cell [9,10]. Extracellular CSP binds to the receptor, ComD, resulting in a phosphorylation cascade that activates the response regulator, ComE. Direct binding of phosphorylated ComE to promoter sequences through the recognition of a pair of direct repeats results in the upregulation of a number of early competence genes including an alternative sigma factor [6]. The regulatory cascade culminates in the activation of over 180 genes [5,6,11,12]. Many genes that encode the antagonistic fratricide effectors are upregulated during competence. These proteins target non-competent cells in the population for lysis, presumably to provide a source of genetic material for DNA exchange [13,14,15,16]. The major competence induced fratricide effectors include the murein hydrolase CbpD, and the bacteriocins CibAB with some contribution by the autolysins, LytA and LytC. In general, only pneumococci that have failed to become competent are lysed by fratricide effectors, because the immunity proteins that provide protection against self-killing are also stimulated during competence and are highly conserved. Pneumococcal populations tend to express one of two dominant CSP signals. This relatively limited repertoire of pherotypes makes cross induction of fratricide immunity between any two co-colonizing strains likely, potentially limiting DNA exchange [17,18,19].

Pneumococcal bacteriocin (pneumocin) production is controlled by the *blp* locus. Like competence, the *blp* locus is stimulated by the accumulation of a peptide pheromone, in this case, called BlpC [20,21]. Pre-BlpC is processed at a double glycine motif and secreted out of the cell via its cognate transporter complex, BlpAB (Fig 1). BlpC binds to and activates the histidine kinase receptor, BlpH, resulting in phosphorylation of the response regulator, BlpR [22]. Phosphorylated BlpR upregulates the 4–6 operons in the *blp* locus including a variety of genes found in the Bacteriocin-Immunity Region (BIR) of the locus [20,23,24]. A version of the *blp* locus has been found in all genome sequenced strains examined to date [22,24]. Unlike the conserved competence system and associated fratricide effectors, the *blp* locus is characterized by significant diversity in peptide pheromone/receptor alleles, pneumocin/immunity content and in the integrity of the BlpAB transporter. There are four major BlpC/BlpH types and at least nine distinct putative structural pneumocins [20,21,22,23,24]. BlpC types are typically restricted in their stimulation of cognate BlpH types, with little evidence of cross stimulation. Additionally, we have shown that nearly 70% of sequenced strains have a disrupted *blpA* gene [22,24]. *In vitro* and *in vivo* studies have shown that *blpA* disrupted strains lack evidence of pheromone secretion and bacteriocin mediated inhibition but retain the ability to respond to exogenous BlpC secreted by surrounding pneumococcal competitors, allowing for protective immunity production encoded in the BIR [24]. These “cheater” strains presumably avoid the energetic cost of self-stimulation and bacteriocin secretion. The success of this strategy for the cheater strain depends on a match between the cheater BlpH receptor and the competitor BlpC, plus a match between cheater immunity and the attacking bacteriocins from the competitor.

Previous work characterizing the competence regulon suggested that there was a regulatory connection between the two systems [6]. If true, pneumocin production during competence would result in the production of antagonistic molecules that would augment the effect of the traditional fratricide effectors. In this study, we demonstrate that CSP can trigger the activation of the *blp* locus in rich media. In the subset of strains with an intact *blpA* gene, this cross talk is primarily controlled by ComE-dependent expression of the *blpABC* operon resulting in increased production and secretion of BlpC through BlpAB. Strains with a disrupted *blpA* gene also demonstrate activation of the bacteriocin locus with the development of competence, but in these strains, the BlpC pheromone is secreted by ComAB and is only noted during the development of competence. Bacteriocin expression increases transformation efficiency in dual culture biofilms suggesting that liberation of DNA through pneumocin predation contributes to the remarkable adaptability of this pathogen.

## Materials and Methods

### Bacterial strains and growth conditions

*Streptococcus pneumoniae* strains and primers used are described in Table 1. *S*. *pneumoniae* strains were grown in Todd-Hewitt broth supplemented with 0.5% yeast extract (THY) or on tryptic soy agar plates supplemented with 5 μg/ml of Catalase (Sigma) or 5% sheep blood Agar plates (SBA) and incubated at 37°C in 5% CO_2_. Starter cultures were prepared for use in broth growth assays by cultivating in THY medium at 37°C to an OD_620_ of 0.5 and then freezing in 20% glycerol. Thawed frozen starter culture were diluted 1/150 into fresh THY media for broth assays unless otherwise noted. C+Y media pH 8.0 and 6.8 were made as previous described [25]. *Escherichia coli* strains were grown in Luria-Bertani (LB) broth or LB agar supplemented with the appropriate antibiotics at 37°C. Antibiotic concentrations used were as follows: for *S*. *pneumoniae*, 500 μg/ml kanamycin, 100 μg/ml streptomycin, 2 μg/ml chloramphenicol, and 200 μg/ml spectinomycin; and for *E*. *coli*, 50 μg/ml kanamycin, 20 μg/ml chloramphenicol, and 100 μg/ml spectinomycin.

**Table 1:** Strain, plasmids, primers and peptides.

**Strains**	** **	**Description**	**Reference**
R6	Unencapsulated laboratory strain; St^s^	R6 wild type	[26]
R6x	R6 with *rpsL*_*K56T*_; St^r^	R6, St^r^	[27]
P271	R6 with kan-*rpsL*+ Janus cassette, Kn^r^, St^s^	R6 with Janus cassette	[26]
P1537	R6 with *comAB*::kan-*rpsL*+, Kn^r^, St^s^	R6 *ΔcomAB*	[28]
P1538	R6 with *comE*::kan-*rpsL*+, Kn^r^, St^s^	R6 *ΔcomE*	[28]
P174	Clinical isolate with *blpA*_*FS*_	Clinical isolate, *blpA*_*FS*_	[24]
PSD100	R6x with intact *blpA*, *BIR*_*p1*_ *-lacZ* reporter; Cm^r^, St^r^,	R6 *BIR*_*p1*_ *-lacZ* reporter	[29]
PSD101	PSD100 with *blpC*::kan-*rpsL*+, Kn^r^, Cm^r^, St^s^	R6 *BIR*_*p1*_ *-lacZ* reporter, Δ*blpC*	[29]
PSD118	PSD100 with *blpH*_*6A*_; Cm^r^, St^r^	R6 *BIR*_*p1*_ *-lacZ* reporter, *blpC*_*R6*_ *H*_*6A*_ chimera	[29]
PSD119	PSD100 with *ΔblpH*; Cm^r^, St^r^	R6 *BIR*_*p1*_ *-lacZ* reporter, *ΔblpH*	this study
PSD120	PSD100 with *blpA*_*FS*_; Cm^r^, St^r^	R6 *BIR*_*p1*_ *-lacZ* reporter, *blpA*_*FS*_	this study
PSD121	PSD120 with *comAB*::*kan-rpsL+*, Cm^r^, Kn^r^, St^s^	R6 *BIR*_*p1*_ *-lacZ* reporter, *blpA*_*FS*,_ *ΔcomAB*	this study
PSD125	PSD100 with *blpC*_*flag*_, Cm^r^, St^r^	R6 *BIR*_*p1*_ *-lacZ* reporter, *blpC*_*flag*_	[29]
PSD127	PSD125 with *ΔhtrA*; Cm^r^, St^r^	R6 *BIR*_*p1*_ *-lacZ* reporter, *blpC*_*flag*_, *ΔhtrA*	[29]
PSD128	PSD125 with *blpA*::kan-*rspL*+; Cm^r^, Kn^r^, St^s^	R6 *BIR*_*p1*_ *-lacZ* reporter, *blpC*_*flag*_, *blpA*::Janus	[29]
PSD129	PSD125 with Δ*blpA*_5–707_; Cm^r^, St^r^	R6 *BIR*_*p1*_ *-lacZ* reporter, *blpC*_*flag*_, *ΔblpA*	[29]
PSD131	PSD125 with *ΔhtrA*, *ΔblpA*; Cm^r^, St^r^	R6 *BIR*_*p1*_ *-lacZ* reporter, *blpC*_*flag*_, *ΔblpA*, *ΔhtrA*	[29]
PSD140	PSD100 with *comAB*::*kan-rpsL+*, Cm^r^, Kn^r^, St^s^	R6 *BIR*_*p1*_ *-lacZ* reporter, *ΔcomAB*	this study
PSD141	PSD100 with *comE*::*kan-rpsL+*, Cm^r^, Kn^r^, St^s^	R6 *BIR*_*p1*_ *-lacZ* reporter, *ΔcomE*	this study
PSD142	PSD118 with *ΔblpA*_*5–707*_; Cm^r^, St^r^	R6 *BIR*_*p1*_ *-lacZ* reporter, *blpC*_*R6*_ *H*_*6A*_ chimera, *ΔblpA*	this study
PSD143	PSD118 with *comAB*::*kan-rpsL+*; Cm^r^, Kn^r^, St^s^	R6 *BIR*_*p1*_ *-lacZ* reporter, *blpC*_*R6*_ *H*_*6A*_ chimera, *ΔcomAB*	this study
PSD144	PSD125 with *comAB*::*kan-rpsL+*, Cm^r^, Kn^r^, St^s^	R6 *BIR*_*p1*_ *-lacZ* reporter, *blpC*_*flag*_, *ΔcomAB*	this study
PSD145	PSD125 with *comE*::*kan-rpsL+*, Cm^r^, Kn^r^, St^s^	R6 *BIR*_*p1*_ *-lacZ* reporter, *blpC*_*flag*_, *ΔcomE*	this study
PSD146	PSD125 with *ΔhtrA*, *comAB*::*kan-rpsL+*; Cm^r^, Kn^r^, St^s^	R6 *BIR*_*p1*_ *-lacZ* reporter, *blpC*_*flag*_, *ΔhtrA*, *ΔcomAB*	this study
PSD147	PSD125 with *ΔhtrA*, *ΔblpA*, *comAB*::kan-rpsL+; Cm^r^, Kn^r^, St^s^	R6 *BIR*_*p1*_ *-lacZ* reporter, *blpC*_*flag*_, *ΔhtrA*, *ΔcomAB*, *ΔblpA*	this study
PSD199	PSD100 with promoter of *blpA*::*kan-rpsL+*; Cm^r^, Kn^r^, St^s^	R6 *BIR*_*p1*_ *-lacZ* reporter, *blpAp*::Janus	this study
PSD200	PSD100 with G-90A at promoter of *blpABC* operon, Cm^r^	R6 *BIR*_*p1*_ *-lacZ* reporter, *blpA*_*pG-90A*_	this study
PSD201	PSD200 with comAB::kan-*rpsL*+, Cm^r^, Kn^r^, St^s^	R6 *BIR*_*p1*_ *-lacZ* reporter, *blpA*_*pG-90A*_, *ΔcomAB*	this study
PSD224	PSD125 with promoter of blpA::kan-*rpsL*+; Cm^r^, Kn^r^, St^s^	R6 *BIR*_*p1*_ *-lacZ* reporter, *blpC*_*flag*_, *blpAp*::Janus	this study
PSD225	PSD125 with G-90A at promoter of *blpABC* operon, Cm^r^, St^r^	R6 *BIR*_*p1*_ *-lacZ* reporter, *blpC*_*flag*_, *blpA*_*pG-90A*_	this study
PSD226	PSD225 with comAB::kan-rpsL+, Cm^r^, Kn^r^, St^s^	R6 *BIR*_*p1*_ *-lacZ* reporter, *blpC*_*flag*_, *blpA*_*pG-90A*_, *ΔcomAB*	this study
D39	Serotype 2	D39 wild type	this study
D39x	D39 *rpsL*_*K56T*_, St^r^	D39 St^r^	this study
P912	19A with *blpI*::kan-*rpsL*+; Kn^r^, St^s^	19A with *blpI*::Janus	this study
P212	19A with Δ*BIR*	19A with non-producing BIR	[24]
P213	19A with *BIR*_*164*_	19A with bacteriocin producing BIR	[24]
PSD299	D39 with *aad9* upstream of *blpT*, BIR_D39_; Sp^r^	D39 with non-producing BIR	this study
PSD300	D39 with *aad9* upstream of *blpT*, BIR_P164_; Sp^r^	D39 with bacteriocin producing BIR	this study
PSD300x	D39 with *aad9* upstream of *blpT*, BIR_P164_; Sp^r^, rpsL_K56T_; St^r^	D39 with bacteriocin producing BIR, St^r^	this study
PSD301	PSD300x with *blpI*::kan-*rpsL*+; Kn^r^, Sp^r^, St^s^	D39 with bacteriocin producing BIR, St^r^, *blpI*::Janus	this study
PSD302	PSD300x with G-90A at promoter of *blpABC* operon, Sp^r^	D39 with bacteriocin producing BIR, St^r^, *blpA*_*pG-90A*_	this study
PSD303	PSD300 with G-90A at promoter of *blpABC* operon, Sp^r^	D39 with bacteriocin producing BIR, *blpA*_*pG-90A*_	this study
PSD304	PSD300 with *comAB*::*kan-rpsL+;* Sp^r^, Kn^r^, St^s^	D39 with bacteriocin producing BIR, *ΔcomAB*	this study
PSD305	PSD300 with *comE*::*kan-rpsL+;* Sp^r^, Kn^r^, St^s^	D39 with bacteriocin producing BIR, *ΔcomE*	this study
PSD306	PSD300 with *BIR*_*p2*_ *-lacZ* reporter. Sp^r^, Cm^r^	D39 with bacteriocin producing *BIR*_*p2*_ *-lacZ* reporter	this study
PSD307	PSD303 with *BIR*_*p2*_ *-lacZ* reporter. Sp^r^, Cm^r^	D39 with bacteriocin producing *BIR*_*p2*_ *-lacZ* reporter, *blpA*_*pG-90A*_	this study
**Plasmids**	**Description**		Reference
pEVP3	Reporter plasmid for integration in *S*. *pneumoniae*; Cm^r^		[30]
pE57	pEVP3 derivative with *BIR*_*p*_ *1-lacZ*; Cm^r^		[29]
pE158	pEVP3 derivative with *BIR*_*p1*_ *-lacZ* and *blpA*_*pG-90A*_; Cm^r^		this study
pE54	pEVP3 derivative with *BIR*_*P2*_ *-lacZ*; Cm^r^		this study
pTOPO	cloning vector plasmid		Invitrogen
pE149	*blpT* and SPD_0465 cloned into TOPO plasmid		this study
**Primers**	**Sequence (5’ to 3’)**	**Description**	** **
1	GATAAAAATGGCTCCTCTGC	F primer used to amplify region of *blpA* and its promoter	
2	TCAAAAATAATTCGCGTCTG	R primer used to amplify region of *blpA* and its promoter	
3	TATTTTATGAAATCTTGAATAGTCATTAAAACTTCTTGAATGGTAAAAAAGTGATTAGAAATTATTTTTTT	F primer used to make site direct mutagenesis of *blpA*_*pG-90A*_	
4	AAAAAAATAATTTCTAATCACTTTTTTACCATTCAAGAAGTTTTAATGACTATTCAAGATTTCATAAAATA	R primer used to make site direct mutagenesis of *blpA*_*pG-90A*_	
5	GGGCCCATGCATGAAGAACATAAGTTTTATCTTT	F primer used to amplify caax with NsiI site	
6	GGCCTCTAGACAGCTGAAAGAAAGACAGCA	F primer used to amplify caax with XbaI site	
7	GAC TTG CCC GGG TCA TTA GCT TTT TTA GTG GA	F primer used to amplify Janus with SmaI site	
8	GAC TTC CCC GGG GAG CAC TTT GTA AGT CTG TTG	R primer used to amplify Janus with Smal site	
9	GATCAATTGCTGGCAGAGGT	F primer used to amplify upstream of blpH	
10	TACCTGACGTGCATACAACAATATCCAAGC	R primer used to amplify upstream of blpH	
11	TTGTATGCACGTCAGGTACTTACTGTGATAC	F primer used to amplify down stream of *blpH*	
12	CTGTAAGATTAAAACTGGAGAAG	R primer used to amplify down stream of *blpH*	
13	GAATTGCCACCT ATATGGAG	F primer used to amplify region of *blpT* and SPD_0465	
14	ATGACAGATACAGACCCCTAC	R primer used to amplify region of *blpT* and SPD_0465	
15	CCG CTC TAG AAC TAG TGG ATC C	F primer used to amplify Sp cassette	
16	CAATTTTTTTATAATTTTTTTAATCTG	F primer used to amplify Sp cassette	
17	TTATCTTTATGGGGTTGATAG	F primer used to amplify region of *blpA* to *blpI* in D39 background	
18	CTTACCAACTTAGTCCCAATTTATCA	R primer used to amplify region of *blpA* to *blpI* in D39 background	
**Peptides**			**Source**
CSP_1_	EMRLSKFFRDFILQRKK		Genscript
BlpC_R6_	GWWEELLHETILSKFKITKALELPIQL		Genscript
BlpC_6A_	GLWEDILYSLNIIKHNNTKGLHHPIQL		Genscript

* Abbreviations used: St: streptomycin, Sp: spectinomycin, Cm: chloramphenicol, Kn: kanamycin.

### Overlay assays

Overlay assays were performed as previously described [27]. Briefly, strains to be assayed for inhibitory activity were spiked into TSA plates supplemented with catalase using small sterile pipet tips. Plates were incubated for 5 hr at 37°C to allow for outgrowth. The sensitive strain, D39x was grown to an OD_620_ of 0.3 to 0.5 and 300 μl was added to a mixture of 100 μl of 1mg/ml catalase, 5 ml of THY and 3 ml of molten TSA. The mixture was carefully poured over the top of the spiked plate, and then incubated overnight at 37°C in 5% CO_2_. Inhibitory activity was indicated by a zone of clearing around the spiked site.

### Construction of site directed mutants, deletion strains and strains used in the biofilm experiments

Primers and peptide sequences are listed in Table 1. To construct single nucleotide mutant in the *BIR*_*p1*_ *-lacZ* reporter strain (PSD100) or the BlpC_FLAG_ expressing background (PSD125), the mutation was first introduced into the original plasmid (pE57) that was used to made the reporter strains by site directed mutagenesis using the Quick Change Kit (Agilent) with primers 3 and 4 [26]. The resulting *blpAp*_*G-90A*_ containing plasmid (pE158) was sequenced to confirm the mutation. To introduce an exchangeable Janus cassette into the *blpA* promoter region, the plasmid pE57 was digested with HpaI to remove a region from *blpA* promoter to the middle of the *lacZ* gene. This product was then ligated with a blunt ended version of the exchangeable Janus cassette (Kn^R^ and St^S^) amplified from strain P271 using primers 7 and 8. The ligated Janus-containing plasmid was transformed directly into PSD100 or PSD125 and selected on kanamycin resulting in strain PSD199 and PSD224. These two strains were then transformed with plasmid pE158 and selected on streptomycin containing media. Exchange of the Janus cassette for the region of interest was confirmed by loss of kanamycin resistance and sequencing. These strains were designated PSD200 (*BIR*_*p1*_ *-lacZ* reporter) and PSD225 (BlpC_FLAG_).

To create deletions in *comAB*, PSD140, and *comE*, PSD141, strains were transformed with cell lysate of P1537 (*comAB*:: *kan-rpsL+*) or P1538 (*comE*:: *kan-rpsL+*)[28] as the *comA* deficient strain or *comE* deficient strain and selected on kanamycin. To construct *blpA* frame shift mutation in the *BIR*_*p1*_ *-lacZ* reporter, PSD120, and *blpA* in frame deletion in the chimera strain, PSD142, the region with the *blpA*::Janus cassette was amplified from strain PSD128 with primers 1 and 2 and transformed into both the reporter and the chimera strain with selection on kanamycin. The mutation was then introduced into these strains by transforming with a fragment of DNA containing the *blpA* frame shift mutation from strain P174 or an unmarked *blpA* deletion amplified from strain PSD129 using the same primers 1 and 2, and selecting on streptomycin as an indication of Janus exchange. The introduction of the deletion was confirmed by PCR. To construct *blpA*_FS_/ΔcomAB dual mutation PSD121, the *blpA*_FS_ reporter strain was transformed with cell lysate of PSD140 containing *ΔcomAB*::Janus and selected on kanamycin.

To construct the *blpH* deficient stain, PSD119, an in-frame deletion DNA fragment was created by PCR sewing using primer pairs 9, 10 and 11, 12. This product was then transformed into a *blpC*::Janus containing strain, PSD101. Because of the close proximity between *blpH* and *blpC* genes, exchange of Janus also removed the *blpH* gene. The introduction of the deletion was confirmed by PCR.

In order to track strains in the biofim model, pneumocin non-producer strain (P212) and producer strain (P213) [24] were cloned to contain a spectinomycin resistance cassette at the 3’ end of the divergently transcribed *blpT* and SPD_0465 genes. The region between the divergently transcribed genes was amplified from R6 with primers 13 and 14 and then cloned into pTOPO vector as pE149 and maintained in *E*. *coli*. The spectinomycin cassette was amplified with primers 15 and 16, and then inserted into pE149 at a unique, blunt ended BstBI site between the *blpT* and SPD_0465 genes. This plasmid was then used to transform P212 and P213 with selection on spectinomycin. A double cross over event resulting in introduction of the spectinomycin cassette was confirmed by PCR. The cell lysate of resulting strains was then transformed into D39 with selection on spectinomycin. This transformation was done to remove the streptomycin resistance marker present in strains P212 and P213 so that transformation events could be tracked. Spectinomycin resistant strains were confirmed by PCR and overlay assays for bacteriocin mediated inhibition or sensitivity. The resulting strains were PSD299 which contains the BIR region derived from D39, which lacks any known bacteriocin genes and carries a disrupted *blpA* gene and strain PSD300, which is a *blp* bacteriocin producer containing the BIR from strain P164 and an intact *blpA* gene [24].

To construct the single nucleotide mutant in the D39 bacteriocin producer (PSD303), a similar strategy to the creation of the *blpAp*_*G-90A*_ mutations in the R6 background was used. In this case, the Janus cassette was inserted into the first bacteriocin gene, *blpI*, in the BIR region, resulting strain PSD301. The mutation was created by exchanging the Janus cassette with the mutated region DNA fragment created by PCR sewing using primer pairs 17, 18 and 3, 4 creating strain PSD302. The entire locus was then moved into a streptomycin sensitive background by transforming PSD302 lysate into D39 and selecting on spectinomycin. Exchange of the bacteriocin locus and the presence of the mutation in the resultant stain PSD303 were confirmed by inhibitory overlay assay, PCR and sequencing.

### Construction of the *BIR*_*p2*_ *-lacZ* reporter for use in bacteriocin expressing strains

The a region of a CAAX protease-like protein in the BIR of strain P164 was amplified from the original P164 using primers 5 and 6 and then cloned into the reporter plasmid, pEVP3, using restriction sites NsiI and XbaI. The resultant *BIR*_*p2*_ *-lacZ* plasmid (pE54) was maintained in *E*. *coli* with chloramphenicol selection and confirmed with DNA sequencing. The plasmid, pE54, was prepared using standard techniques and transformed into PSD300 or PSD303 with selection on chloramphenicol. The *BIR*_*p2*_ *-lacZ* reporter activity of resultant strains, PSD306 and PSD307, was verified by a blue color change when plated on x-gal plates supplemented with 500 ng/ml BlpC_R6_ inducer.

### Dose-response assay and peptide induction assays

Pneumococcal *lacZ* reporter strains were grown in THY to an OD_620_ of 0.1. For some assays, cultures were first preinduced with 200ng/ml of CSP_1_ for 10 minutes prior to stimulation with BlpC. Cultures were dispensed in 90 μl aliquots into a 96 well plate pre-loaded with 10 μl of serial dilutions of synthetic BlpC_R6_ between 2 μg/ml and 0 ng/ml. Miller Units were determined after incubation for 1 hr at 37°C. For dose-response curves, samples were analyzed in triplicate, peptide concentrations were converted to log values and the response for each strain was normalized between 100 and 0%. Dose-response curves were generated using nonlinear regression with Prism 6.0. For peptide induction, 200 ng/ml CSP_1_ was added to cultures at an OD_620_ of 0.1. Cultures were sampled every 30 min for 3 hours and assessed for OD_620_ and Miller Units at each time point. Uninduced samples were run simultaneously as controls. Each assay was performed at least three times, representative assays are shown.

### Miller assay

For time course experiments, 100 μl samples were collected at the indicated time points in triplicate and lysed in a 96 well plate by the addition of 1 μl 10% Triton X-100. Plates were stored on ice until completion of the assay. At the end of the assay, plates were incubated at 37°C for 10 min and examined for complete lysis of cells. A 25 μl volume of ONPG in 5× Z-buffer (5 mM MgCl, 50 mM KCl, 0.3 M Na_2_HPO_4_, 0.2 M NaH_2_PO_4_, 250 mM β-mercaptoethanol, 4 mg/ml ONPG) was added, and the reaction was allowed to continue until a color change was visible or for 60 min if no color change was detected. A 50 μl volume of 1 M NaCO_3_ was added to stop the reaction, and plates were read at OD_405_. Miller units were determined as previously described [29].

### BlpC secretion using chimeric strains

Pneumococcal BlpC_R6_H_6A_ chimera strains were grown to an OD_620_ of 0.2 and split into three 1ml cultures for untreated, treated with 200 ng/ml BlpC_6A_ or treated with 200 ng/ml CSP_1_. After 30 minute incubation at 37°C, Miller units were calculated on 100 μl of cells and the remainder of the culture was pelleted and the supernatant filter sterilized. Supernatants were used to determine secreted BlpC_R6_ concentrations by adding 50 μl of supernatant to 50 μl of a *blpC* deleted BlpC_R6_-responsive reporter strain (PSD101) and incubating for 1hr at 37°C. Cells were lysed and Miller Units were calculated as above. BlpC concentrations were determined by interpolating this value using the dose-response of the reporter strain to known concentrations of synthetic peptide BlpC_R6_ [26]. Unstimulated supernatant and blank medium were used as controls.

### Western blot analysis

Pneumococcal strains expressing BlpC_FLAG_ were grown to an OD_620_ of 0.2 and divided into untreated or treated with 200 ng/ml BlpC_R6_, 200 ng/ml CSP_1_, or both peptides. At indicated time points, 1.5 ml of pelleted cells was resuspended in 40 μl of Tricine Sample Buffer (Bio-Rad) and boiled for 5 min. The same procedure was used for dose-response samples except that increasing concentrations of BlpC were used to induce the cultures. For time points after CSP induction pellets were lysed in Cell Lytic Buffer B (Sigma) and protein concentrations determined by Bradford Assay to allow for equal loading. Proteins were separated using 16.5% Tris-Tricine gel (Bio-Rad) and transferred onto 0.45 μm PVDF membrane (Immobilon). Membranes were dried overnight at 37°C and fixed in 5% paraformaldehyde for 30 min at 37°C prior to blocking with 5% milk-TBS solution [30]. Primary antibodies used were anti-FLAG M2 antibody (Stratagene) at 1:2000 dilution or anti-pneumolysin (Thermo) at 1:2000 dilution as a loading control. HRP-conjugated goat anti-mouse antibody was used as a secondary antibody at 1:10,000. Quantification was performed using AlphaView (Protein Simple) and loading was normalized using the pneumolysin band. Signals for both antibodies were confirmed to be in the linear range for quantification by analyzing quantification data of serial dilutions of samples. For analysis of the *blpAp*_G-90A_ mutant compared with wildtype, the results of three blots were used for analysis.

### Analysis of the BlpR binding site in publically available pneumococcal genomes

The *blpA* promoter region of 229 publically available pneumococcal genomes that were available in contig assemblies was analyzed. The list of strains analyzed has been previously reported [22]. The sequences were individually searched for the presence of the ACCATTCAG sequence at -90 position from the start codon of *blpA* gene.

### Biofilm model

Establishment of a biofilm was performed as previous described [31]. Briefly, pneumococcal D39 derivative cultures were diluted 1:25 into chemically defined media (CDM), grown to an OD_620_ of 0.5, then further diluted 1:500 into pre-warmed 34°C CDM. Pneumocin producer (PSD300 Sp^R^), pneumocin producer with the *blpA*_*pG-90A*_ mutation (PSD303 Sp^R^) or non-producer (PSD299Sp^R^) were co-inoculated with the sensitive (D39x St^R^) strain in a 500 μl volume and allowed to form a biofilm on confluent human epithelial H292 cells that had been fixed to glass coverslips in 24 well plates. The biofilm was grown at 34°C in 5% CO_2_. Growth media was changed every 12 hours for 3 days. To assess the biofilm population, each well was washed with PBS three times and the samples were maintained in 500 μl of PBS. Plates were sealed with parafilm and sonicated for 12 seconds in a sonicator bath (Ultrasonic cleaner, Sper Scientific) to disperse bacteria from the biofilm. Bacterial biomass and compositions of the biofilms were determined by serial dilutions of viable colony forming unit (CFU) counts on single or dual selective plates. Competitive index was calculated by dividing the output ratio to of the co-inoculated strains to the input ratio. Transformation efficiency was determined in wells with transformation events by the percentage of dual^R^ counts over the total (Sp^R^ + St^R^—dual^R^) counts. Two-tailed Mann-Whitney tests were performed to determine statistical significance on data as indicated.

## Results

### CSP induces the *blp* locus through a ComE dependent process

**Fig 2:**
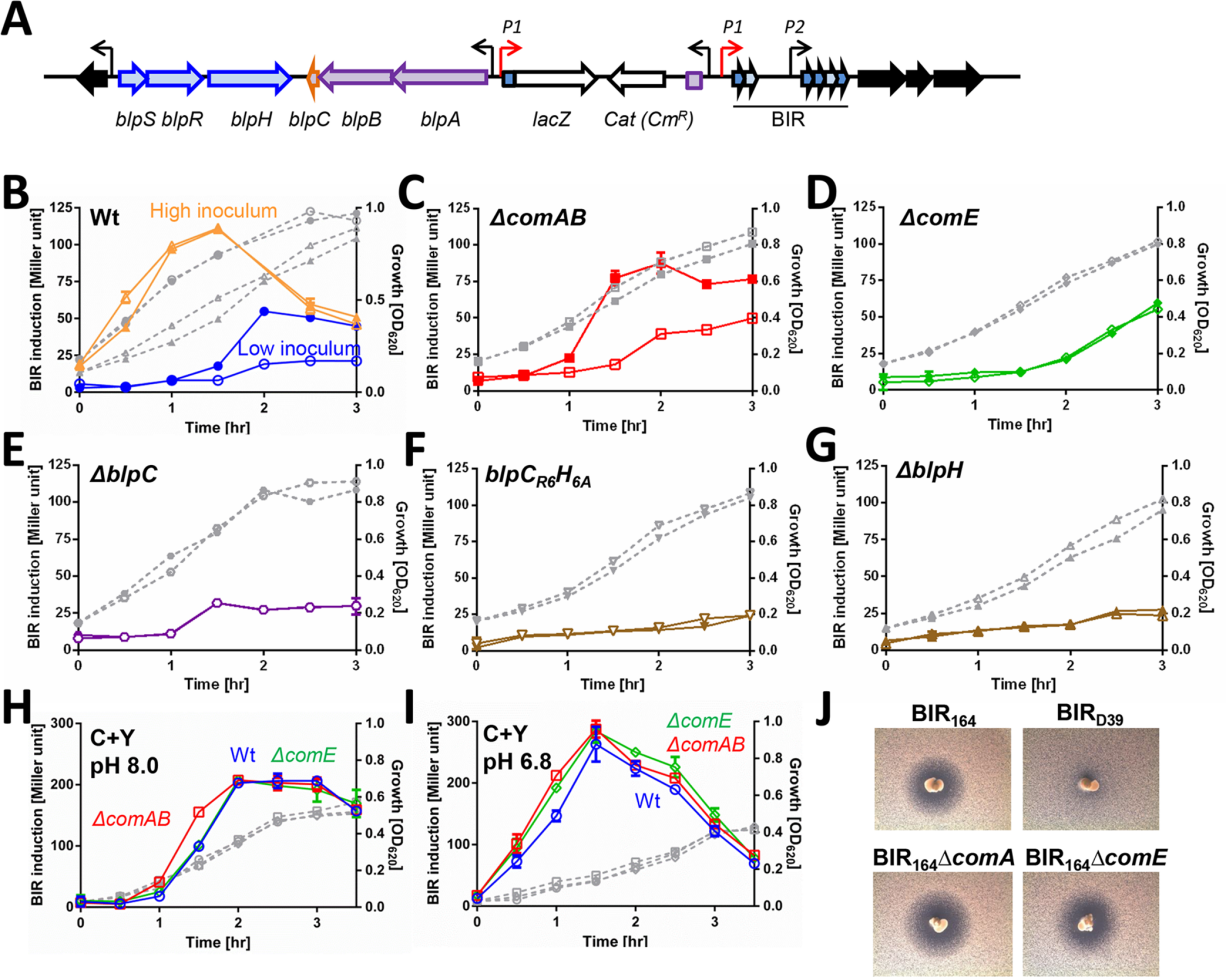
CSP induction of *BIR* transcription is dependent on ComE, BlpH and BlpC but not ComA. *BIR*_*p1*_ *-lacZ* reporters or D39 derivatives expressing inhibitory pneumocins were used to assess the response of the *blp* locus to competence induction. (A) Diagrammatic representation of the *BIR*_*p1*_ *-lacZ* reporter fusion. This strain carries an integrated reporter plasmid in which the 5’ most BIR promoter (P1) is driving *lacZ*. A second BIR promoter is noted as P2. Integrated plasmid sequences are shown as white arrows. The gene designations are shown below the arrows. Derivatives of this reporter fusion were used to assay for BIR response to CSP induction in THY (B-G) or C+Y (H-I), Open symbols represent samples that were not induced with CSP, closed symbols represent cultures that were induced with 200 ng/ml CSP at time 0. Gray dashed lines denote the cellular growth. Comparison of BIR transcriptional response to CSP addition in Wt PSD100 (B), *ΔcomAB* PSD140(C), *ΔcomE* PSD141(D), *ΔblpC* PSD101(E), *blpC*_*R6*_ *H*_*6A*_ chimera PSD118(F) or *ΔblpH* PSD119(G) strains. Comparison of Wt, ComAB and ComE deficient reporters in natural BIR induction in competence permissive C+Y pH 8 (H) or competence non-permissive C+Y pH 6.8 (I). (J) Results of an inhibitory overlay assay using D39 pneumocin producing strains PSD300 (BIR_164_)_,_ PSD304 (BIR_164_ *ΔcomAB*), PSD305 (BIR_164_ *ΔcomE*) and the pneumocin non-producer, PSD299 (BIR_D39_).

Previously, Peterson *et al* had shown using microarray analysis that the gene encoding the bacteriocin/pheromone transporter, *blpA*, was one of the 13 genes or operons induced by CSP early during competence development [6]. The group did not note upregulation of the remainder of the *blp* locus, including the genes encoding *blp* regulatory proteins or the inhibitory pneumocins and their associated immunity proteins. This experiment was performed in the D39 background, a strain that encodes a disrupted, non-functional version of the BlpA transporter. We hypothesized that if *blpABC* transcription but not BIR transcription is induced by CSP addition, significant induction of the bacteriocin genes may only be observed in the 30% of the pneumococcal population that have an intact BlpA transporter and retain the capacity to secrete BlpC allowing for auto amplification of the *blp* locus. To determine if CSP addition can result in upregulation of the entire *blp* locus in an intact BlpA background, we examined the effect of addition of synthetic CSP on a variant of the R6 strain laboratory strain that was engineered to contain an intact *blpA* gene (PSD100). In addition to an intact *blpA* gene, this strain also contains an integrated reporter plasmid in which *lacZ* expression is driven by the 5’-most BIR promoter (Fig 2A red arrow). Initial experiments examining competence-bacteriocin cross induction were performed in THY media where natural competence has not been previously observed. We found that the degree of locus activation is dependent on the starting inoculum; low starting inocula (1:150) do not show significant spontaneous activation of the locus during growth in THY, while a three-fold increase in inoculum demonstrates robust activation (Fig 2B). PSD100 grown at high and low inocula was induced with 200 ng/ml of CSP_1_ during early growth (at an OD_620_ of 0.1) and transcriptional activity of the BIR promoter was compared with uninduced samples. As shown in Fig 2B, CSP addition to the low inoculum samples resulted in a notable increase in BIR transcription compared with uninduced samples at 1.5 hours after addition and peaking at 2 hours after addition. CSP did not induce BIR transcription above what was noted in the uninduced cultures in the high inoculum samples. To determine the requirements for CSP dependent BIR activation noted in the low inoculum samples, the induction kinetics of PSD100 derivatives deficient in the competence transporter, *comAB* (Fig 2C), the competence regulator, *comE* (Fig 2D), and the *blp* pheromone, *blpC* (Fig 2E) were investigated in cultures that were grown from a low starting inoculum. No BIR transcription was noted in the *comE* or *blpC* deletions strains, but the response to CSP was preserved in the *comA* deletion background. These results demonstrated that CSP activation of the BIR promoter requires the competence regulators and the bacteriocin pheromone, consistent with a ComE mediated increase in *blpABC* transcription. To verify that a BlpC/BlpH interaction was required for CSP dependent BIR induction, we determined the response in a PSD100 derivative expressing a mismatched BlpC/BlpH pair. This chimeric strain expresses an intact and functional BlpH_6A_ that cannot bind to its encoded BlpC_R6_ pheromone [26]. Consistent with the requirement for BlpC/H interaction for CSP dependent BIR induction, the chimeric strain failed to demonstrate any BIR transcription in response to CSP addition (Fig 2F). Similarly, a strain carrying an in frame deletion of *blpH* also did not show BIR activation in response to addition of CSP (Fig 2G). These data demonstrate that CSP induction of BIR transcription in rich media occurs via CSP acting through ComD/E rather than as a result of a direct interaction of CSP with the BlpH receptor. In addition, the fact that the CSP transporter, ComAB, was dispensable demonstrates that the induction is not due to ComAB augmented BlpC secretion. The requirement for ComE and BlpC in combination with the one to one and a half hour delay in our ability to detect a noticeable increase in BIR transcription is consistent with the hypothesis that CSP induces ComE mediated transcription of the *blpABC* operon resulting in more rapid accumulation of extracellular BlpC which can then go on and auto-induce the locus through binding to and activation of BlpH.

### Natural induction of the *blp* locus is independent of competence in broth and plate grown organisms

Because our initial experiments by-passed the spontaneous development of competence with the addition of exogenous CSP_1_, the role of natural competence in *blp* induction was not assessed. To determine if natural induction of the *blp* locus requires the development of spontaneous competence, reporter strains with or without a deletion in the genes encoding the CSP transporter, *comAB*, or the competence regulator, *comE* were grown in competence permissive C+Y media at pH 8 (Fig 2H) or competence non-permissive C+Y media at pH 6.8 (Fig 2I). We reasoned that if competence induction is required for activation of the *blp* locus in broth, then we would expect activation of the locus to occur only under competence-permissive conditions and in the presence of an intact competence transporter or regulator. Although the degree of induction of the BIR reporter differed in the two media types, robust spontaneous BIR activation was observed under both competence permissive and non-permissive conditions, even when low starting inocula were used. In addition, no differences were noted between the wildtype, *comAB* or *comE* deletion reporter strains. These data combined suggest that natural competence is not required for induction of the *blp* locus, at least under the conditions tested.

To examine whether the competence system is required for *blp* mediated inhibition noted in overlay plates, active pneumocin secreting strains with and without deletions in *comAB* and *comE* were tested for inhibition against a sensitive overlay strain (Fig 2J). Consistent with data from broth grown organisms, *blp* mediated inhibition was preserved in deletion strains demonstrating that inhibition in plate grown organisms does not require an active competence system.

### CSP induces production and enhances secretion of BlpC

**Fig 3:**
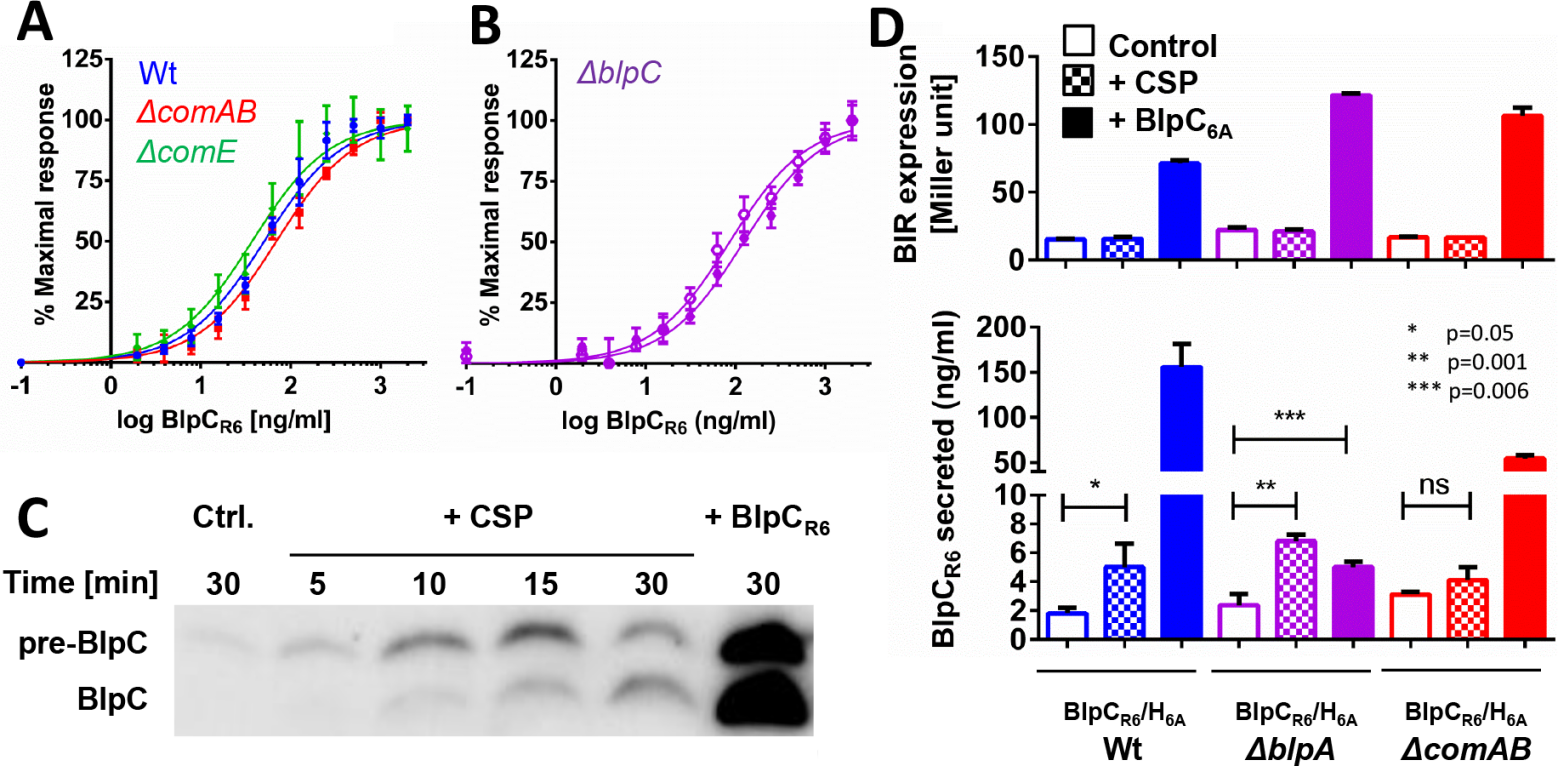
CSP induces production and secretion of BlpC. (A) BlpC_R6_ dose response in wt PSD100 (blue), *ΔcomAB* PSD140 (red), *and ΔcomE* PSD141 (green) version of *BIR*_*p1*_ *-lacZ* reporter strains. (B) BlpC_R6_ dose response in a *ΔblpC* reporter strain PSD101 (purple open) and following 10 minutes of CSP pretreatment (purple closed). (C) Western blot of cell lysate of BlpC_FLAG_ strain (PSD125) after treatment with CSP or BlpC_R6_. Equal amounts of protein were loaded into each lane. (D) BlpC_R6_ secretion assay using chimeric BlpC_R6_/H_6A_ (BlpC_R6_ secretor, BlpC_6A_ responsive) *BIR*:*lacZ* reporter strains: PSD118 (blue), PSD142 (*ΔblpA*, purple), and PSD143 (*ΔcomAB*, red). Upper: *BIR*_*p1*_ *-lacZ* induction of chimera strains 30 minutes after peptide addition. Lower: BlpC_R6_ concentration secreted into supernatant 30 minutes after induction. P values as noted were determined by student T-test.

To examine whether competence-mediated BIR induction might be due to an enhanced response to BlpC rather than an increase in the local concentration of the peptide, we performed a dose-response assay comparing the response of the wildtype, *ΔcomAB* and *ΔcomE* deletion mutants of the BIR reporter strain to increasing concentrations of synthetic BlpC (Fig 3A). To address whether CSP treatment itself changes the response to BlpC, we performed the same assay on a Δ*blpC* deletion strain 10 minutes after the addition of CSP (Fig 3B). There were no differences in the dose-response curves in any of the experimental conditions tested suggesting that CSP treatment or the presence of an intact competence system does not enhance the responsiveness of the strain to exogenous BlpC.

The requirement for BlpC in the CSP-mediated enhancement of BIR transcription suggested that CSP increases BlpC secretion through upregulation of the *blpABC* transcript. To demonstrate the effect of CSP addition on BlpC levels, we induced a reporter strain carrying a FLAG-tagged version of BlpC with CSP. We have shown previously that BlpC_FLAG_ bands are induced after addition of synthetic BlpC and are only seen in BlpC_FLAG_ containing strains. The BlpC_FLAG_ expressing strains are functional BlpC knockouts because the tagged version of the peptide is processed by BlpA but the peptide is not secreted and remains with the cell pellet [26]. Using this strain we showed that CSP addition resulted in the accumulation of BlpC_FLAG_ that was detectible as early as 5 minutes after induction (Fig 3C) demonstrating that CSP addition increases the production of BlpC peptide.

Because BlpC_FLAG_ is not secreted [26], we could not confirm that CSP-induced BlpC production results in secretion of functional BlpC. To examine this further, we used the *BIR*_*p1*_ *-lacZ* reporter strain expressing a chimeric or mismatched BlpC_R6_/H_6A_ pair shown in Fig 2F. This strain responds to a different type of BlpC pheromone than it secretes [26]. When treated with exogenous BlpC_6A_, the *blp* locus, including the BIR (Fig 3D top) and *blpABC* promoters are stimulated and the strain secretes BlpC_R6_ (Fig 3D bottom) [26]. Because the secreted BlpC_R6_ cannot activate the BlpH_6A_ expressed by the chimera [22,26], the use of this strain allows us to examine just the newly synthesized and secreted pheromone upon CSP addition while the contribution from the BlpH-mediated, positive feedback that is derived from new BlpC production is excluded. To evaluate for small changes in BlpC secretion, the assay was performed in strains that are deficient in the outer surface protease, HtrA. We have previously shown that HtrA deficient chimeric strains secrete significantly more BlpC than HtrA sufficient strains [26]. Addition of CSP to the BlpC_R6_/H_6A_ chimera did not induce the BIR promoter in the chimera strains, consistent with our previous observations (Fig 2F and Fig 3D top). CSP addition did result in a small but noticeable increase in BlpC secretion over uninduced control samples within 30 minutes of CSP addition (Fig 3D bottom). The increase was considerably less than that noted following BlpC_6A_ stimulation. These results confirm that CSP stimulation results in secretion of BlpC, but also demonstrates that CSP stimulation of the *blpABC* operon is significantly less efficient in promoting this BlpC secretion than the stimulation promoted by BlpC itself.

### CSP stimulation of the *blpABC* operon is mediated by ComE-cross recognition of a BlpR binding site

**Fig 4:**
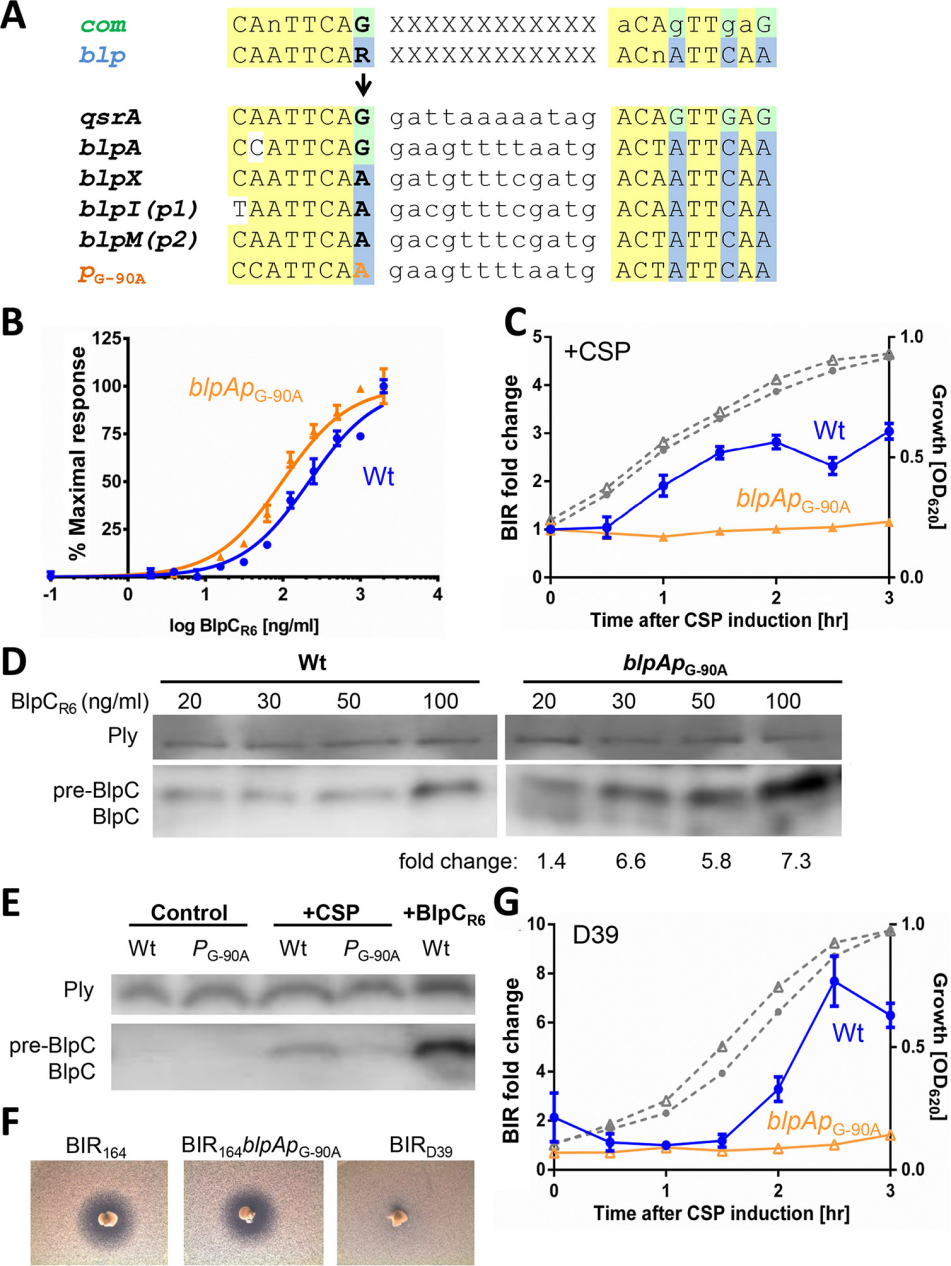
ComE-like binding site in the *blpABC* promoter is required for CSP induction of BlpC secretion. (A) Sequence analysis of *blp* regulated promoters. Consensus sequences that support ComE or BlpR binding are shown on the top of the alignment with conserved nucleotides as capital letters. Alignment of identified BlpR binding sites from the TIGR4 *blp* locus promoters was adopted from [32]. A guanine in the *blpA* promoter binding site at -90 from the start codon is labeled with an arrow and represents the only BlpR binding site within the *blp* locus with this change. The *qrsA* promoter also has a guanine at this position; this promoter is known to be regulated by both ComE and BlpR. Gene designations identify the first gene in the operon that is controlled by the promoter, P1 and P2 refer to the BIR promoters noted in Fig 2A. The created *blpAp*_G-90A_ sequence is shown with the nucleotide change in orange. Shared nucleotides between the two consensus sequences and the listed binding sites are highlighted in yellow. Nucleotides that differ between the two are highlighted in either green (ComE) or blue (BlpR). (B) BlpC_R6_ dose response assay using *BIR*_*p1*_ *-lacZ* reporter strains: PSD100 (Wt, blue circle), and PSD200 (*blpAp*_G-90A_, orange triangle). (C) CSP induction assay using *BIR*_*p1*_ *-lacZ* reporters, PSD100 and single nucleotide mutant at *blpA* promoter PSD200 (*blpAp*_G-90A_). CSP induced fold change in BIR transcription normalized to uninduced samples is shown. Gray dashed lines denote the cellular growth. (D) Western blot of cell lysates of BlpC_FLAG_ strains PSD125 (Wt) and PSD225 (*blpAp*_G-90A_) after treatment with increasing concentrations of BlpC_R6_. Unprocessed BlpC_R6_ is denoted as pre-BlpC. Fold change compared with wildtype levels for each concentration is shown after values were normalized with pneumolysin (Ply) loading control. (E) Western blot of cell lysates of BlpC_FLAG_ strains PSD125 (Wt) and PSD225 (*blpAp*_G-90A_) after treatment with CSP or BlpC_R6_. Unprocessed BlpC_FLAG_ is denoted as pre-BlpC. (F) Inhibitory overlay assay using D39 pneumocin producing strains, PSD300 (BIR_P164_), PSD303 (BIR_P164_ *blpAp*_G-90A_), and non-producing PSD299 (BIR_D39_) strain. (G) Response to CSP induction in D39 background *BIR*_*p2*_ *-lacZ* reporter strains. CSP induced fold change in BIR transcription normalized to uninduced samples is shown for PSD306 (Wt) and single nucleotide mutant at *blpA* promoter PSD307 (*blpAp*_G-90A_). Gray dashed lines denote the cellular growth.

We have shown that CSP stimulation of the BIR promoter requires ComE and is mediated by early accumulation of BlpC in the media. We hypothesized that ComE may bind to and upregulate the *blpABC* promoter during the development of competence. Comparison of the BlpR consensus sequence preceding the *blpABC* operon with other *blp* promoters and the known ComE consensus sequence [12,20,21,32] demonstrated a single nucleotide within the 5’ binding site that could support ComE binding (Fig 4A). The guanine at position -90 from the start codon of *blpA* (Fig 4A arrow) is found in known ComE binding sequences and is conserved in the promoter of the *qrsA* gene, which was previously shown to be controlled by both ComE and BlpR [32]. All other BlpR regulated *blp* promoters including the P1 and P2 promoters in the BIR that drive bacteriocin-immunity production and the promoter preceding the presumed immunity gene operon *blpXYZ* have an adenosine at this position. To determine if this nucleotide was responsible for ComE-mediated upregulation of the *blp* locus, we created a single nucleotide variant reporter strain with a G to A mutation at site -90, *blpA*_*pG-90A*_ (Fig 4A, orange font). This strain responds to exogenous BlpC with a similar dose response curve to the wildtype strain suggesting that the nucleotide change does not affect BlpR binding to the BIR promoter (Fig 4B). When the single nucleotide variant reporter was assessed for the response to CSP stimulation, we found that *BIR* transcription in this strain is no longer induced by CSP (Fig 4C). To better assess the effect of the mutation on BlpC production in response to CSP or BlpC addition, the *blpAp*_G-90A_ mutation was also studied in the BlpC_FLAG_ producing background. When treated with a range of different concentrations of BlpC, we found that in all cases, production of BlpC_FLAG_ is increased in the mutant *blpAp*_*G-90A*_ background compared with the wildtype (Fig 4D). Consistent with the lack of CSP induced BIR transcription in the *BIR*_*p1*_ *-lacZ* background, CSP induction produces significantly less BlpC_FLAG_ in strains carrying the *blpAp*_*G-90A*_ mutation compared with the wildtype BlpC_FLAG_ strain (5.4+/- 2.6 fold decreased compared with wildtype) (Fig 4E). These data are consistent with the hypothesis that the *blpABC* promoter supports ComE binding due to a single nucleotide change from the consensus BlpR binding site. To ensure that this single nucleotide variant mutation does not affect bacteriocin secretion, we performed inhibitory overlay assay using pneumocin producing strains (D39 with BIR_P164_). Overlay assays with the bacteriocin producing strain carrying the *blpA*_*pG-90A*_ mutation did not affect the ability of the strain to inhibit a sensitive overlay strain, demonstrating that the competence independent pneumocin secretion is intact in this strain (Fig 4F). This is consistent with our findings in the *comA* or *comE* mutants in this background (Fig 2J). To confirm the effect of the mutation on CSP mediated stimulation of the BIR in the pneumocin secreting D39 backgrounds, a separate BIR_*P2*_ *-lacZ* reporter was created in the D39 derivatives carrying the BIR_P164_. In these strains, the *lacZ* gene is being driven by the P2 promoter in the BIR of the pneumocin producing strains. These reporter strains demonstrated the same dependence of CSP mediated BIR upregulation on the presence of the guanidine residue at the -90 location as was noted in the R6 *BIR*_*p1*_ *-lacZ* derivatives (Fig 4G).

To determine if the BlpR/ComE binding site in the promoter of *blpA* is conserved in the pneumococcal population, we examined the *blpA* promoter in 229 publically available pneumococcal genomes [22]. An identical sequence was identified at the -90 location in all strains suggesting that the capacity for cross talk is highly conserved.

### ComA processes and secretes BlpC

**Fig 5:**
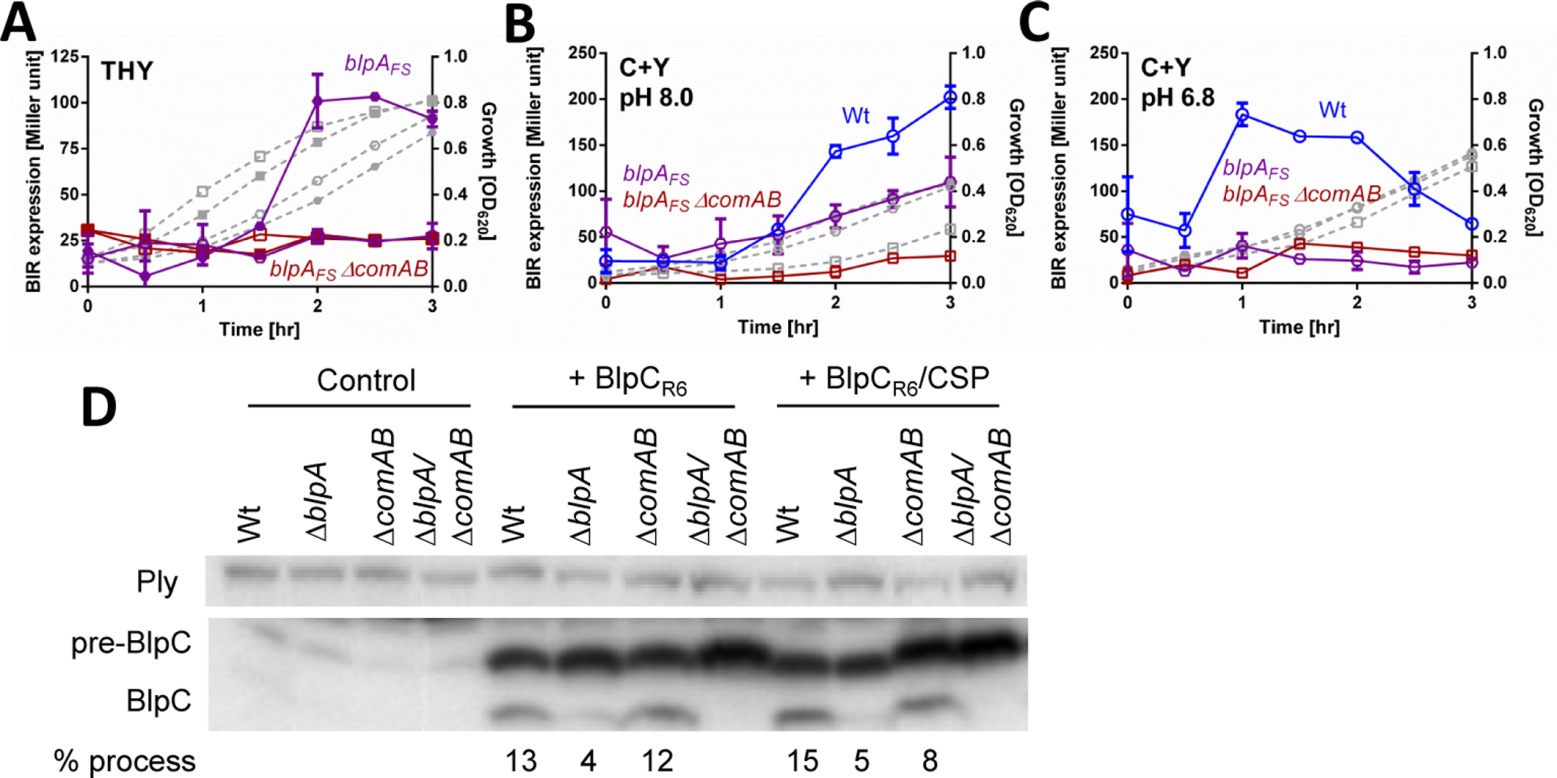
ComA can process and secrete BlpC independently of BlpA. (A-C) *BIR*_*P1*_ *-lacZ* transcriptional response in wildtype (blue circles), *blpA*_*FS*_ (purple octagon) and *blpA*_*FS*_/*comAB* (red squares) mutant reporters during growth in broth. Closed symbols denote CSP addition, open symbols denote no peptide added. Cellular growth is shown as grey lines. (A) Response to CSP added at time 0 in THY. (B-C) Natural *BIR*_*P1*_ *-lacZ* induction in competence permissive (B) and competence non-permissive C+Y media (C). (D) Western blot of cell lysate of BlpC_FLAG_ expressing strains in *ΔhtrA* background: PSD127 (Wt), PSD131 (*ΔblpA*), PSD146 (*ΔcomAB*) and PSD147 (*ΔblpA/comAB*) after treatment with CSP or BlpC_R6_ or both BlpC_R6_/CSP. Preprocessed BlpC_FLAG_ is denoted as pre-BlpC, the processed form is BlpC. Anti-pneumolysin (Ply) antibody was used as a loading control. Densitometry analysis was performed to determine % processed BlpC_FLAG_ of the total BlpC_FLAG_ detected in each strain.

We noted while performing experiments with the a BlpC_R6_/H_6A_ chimera strain that *comAB* deficient chimeras secreted less BlpC_R6_ in response to BlpC_6A_ stimulation when compared with wildtype chimeras (Fig 3D bottom, solid blue vs solid red bar) despite similar up regulation of the BIR region in response to peptide addition (Fig 3D top). To examine this further, we evaluated BlpC secretion using wild type, *ΔblpA*, and *ΔcomA* deletion version of blpC_R6_H_6A_ chimera strains. Consistent with our previous findings, chimera strains with an in-frame, unmarked deletion in the *blpA* gene were noted to secrete a small but detectible quantity of BlpC_R6_ following induction with BlpC_6A_ when compared with uninduced controls (Fig 3D bottom, open purple and solid purple bars). In this assay, we also noted that CSP-stimulated BlpC secretion seems to require *comA* because little to no secretion of BlpC in response to CSP is seen in Δ*comAB* mutants but wildtype levels are secreted by Δ*blpA* mutants (Fig 3D bottom). This observation suggested that ComAB may have the capacity to secrete some BlpC. Although there was no appreciable defect in CSP response in the *BIR*_*P1*_ *-lacZ* reporter with a deletion in *comAB* (Fig 2C, 2H and 2I) suggesting that there was no contribution of ComA to the CSP-mediated stimulation in broth, the chimera data suggested that the contribution of ComA may be more apparent in strains that lack a functional BlpA. To assess this possibility, we created a *BIR*_*P1*_ *-lacZ* reporter strain with the same frame shift mutation in *blpA* (*blpA*_*FS*_) that is found in the majority of cheater strains to determine if ComA-mediated BlpC secretion in this background was sufficient to stimulate the BIR promoter. Surprisingly, this strain showed similar BIR induction in response to CSP addition as the wildtype or *comA* deletion strains (Fig 5A and Fig 2B and 2C). A *blpA*_*FS*_ /Δ*comA* dual mutant reporter no longer responded to CSP with BIR induction consistent with ComAB mediated BlpC secretion in the blpA_FS_ background. These data suggest that the secretion of BlpC can be mediated by both BlpA and ComAB transporters. To determine if the *blpA*_*FS*_ reporters would demonstrate natural *BIR* induction in response to the development of competence independent of CSP addition, we compared the response of the *blpA*_*FS*_ *BIR*_*P1*_ *-lacZ* reporter in competence permissive and non-permissive media. In these assays, strains were allowed to grow and BIR transcription was assessed without the addition of any peptide pheromones. Consistent with a requirement for competence stimulation for BIR induction in the *blpA* mutant strain, the *blpA*_*FS*_ reporter strain only activated BIR transcription in competence permissive media while no induction was seen in non-permissive broth (Fig 5B and 5C). The *blpA*_*FS*_ /Δ*comA* double mutant did not demonstrate BIR induction in either media. This finding differentiates strains lacking *blpA* from those lacking *comAB* because the *comAB* deficient strain retains spontaneous activation even in non-permissive media (Fig 2I). To further quantify and define the relative contribution of the two transporters to BlpC secretion, we examined BlpC_FLAG_ processing in *blpA* deficient, *comA* deficient and dual *blpA/comA* knockout strains. Similar to the chimera experiments, HtrA deficient strains were used for this assay so that BlpC processing could be more easily detected [26]. The BlpC_FLAG_ is expressed in two forms in the wildtype strain, a longer form that represents the unprocessed peptide and a smaller form that represents the processed peptide in which the signal sequence has been removed. We had previously used these strains to show that *blpA* was required for BlpC_FLAG_ processing [26]. We found that fixation of western blot membranes after transfer significantly increased the overall sensitivity of the assay [30]. Upon BlpC induction, a small but notable amount of processed BlpC_FLAG_ could still be detected in Δ*blpA* deletion strains (Fig 5D). This processed band was lost in the *blpA/comA* double mutants consistent with the contribution of ComAB to BlpC processing. Densitometry of western blots demonstrated that, in the absence of BlpA, ComA can process approximately 30% of the processed amount shown in the *comA/blpA* sufficient wildtype strain (Fig 5D). The amount of BlpC processing in wildtype and ComAB deficient strains was indistinguishable following induction with BlpC alone, suggesting that in the presence of BlpAB, processing preferentially occurs through the BlpAB complex. When BlpC_FLAG_ expressing strains were stimulated with both BlpC and CSP, the contribution of ComA to overall processing was more readily apparent, although the difference between the wildtype and *blpA* mutant strain was still significant. Although the contribution of ComAB to stimulation of the *blp* locus through secretion of BlpC is not seen in broth culture or in plate overlay assays in strains with an intact *blpA* gene, it seems to enhance the effect of CSP stimulation allowing for more rapid accumulation of BlpC, under conditions where pre-existing BlpA levels are low or absent.

### Pneumocin production promotes competition and co-stimulation with CSP promotes DNA exchange in mixed biofilms

Induction of the *blp* locus during the development of competence may have a number of beneficial outcomes including increased liberation of DNA from surrounding strains. This is particularly true because competence induced fratricide effectors and their associated immunity proteins are highly conserved in pneumococcus while the *blp* bacteriocins are more variable. This variability may allow for a broader range of predation compared with that promoted by fratricide alone. To examine the impact of competence/pneumocin cross talk on DNA exchange, we utilized the biofilm model developed by Marks *et al* [31]. This model was chosen because, unlike other pneumococcal biofilm models, it was shown to support DNA exchange through activation of the competence system but does not require exogenous addition of CSP for either biofilm establishment or for detectible transformation events. This advantage allowed us to examine the effect of competence and bacteriocin cross talk on competition and transformation during natural induction of both loci. For this assay, we introduced spectinomycin resistance into the pneumocin producing derivative of D39 (BIR_164_), a *blpAp*_G-90A_ mutant of this strain (BIR_164_ *P*_*G-90A*_) and an otherwise isogenic pneumocin non-producer (BIR_D39_) for use as competitor strains. The inhibitory phenotypes of these strains are shown in Fig 4G. Biofilms were co-inoculated with one competitor strain and a streptomycin resistant donor strain that carried the non-functional D39 *blp* locus.

**Fig 6:**
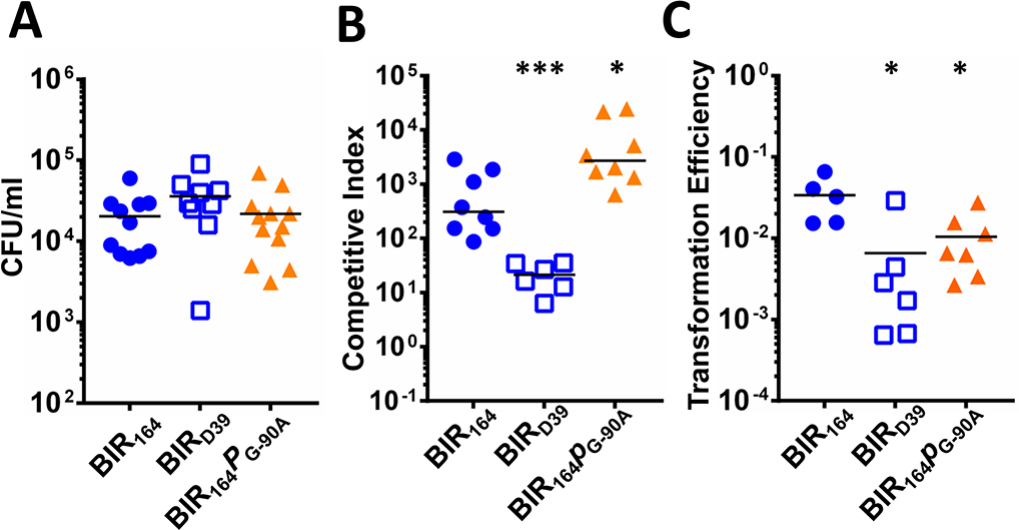
Pneumocin production promotes competition and DNA exchange in pneumococcal biofilms. (A) Biomass of biofilms when inoculated with single competitor strains: pneumocin producers PSD300 (Wt, BIR_164_, blue circle) or PSD303 (BIR_164_ *blpAp*_G-90A,_ orange triangle), and non-producer control PSD299 (BIR_D39_, blue square). (B-C) PSD300, PSD303 or PSD299 were co-inoculated with sensitive strain, D39x at ratio of 1:10 to form biofilm. Three day old biofilms were disrupted and plated on single selective media to determine (B) competition, and dual selective media to examine (C) transformation efficiency. Two-tailed Mann-Whitney tests were performed to determine statistical significance. * denotes *p*<0.05, *** denotes *p*<0.001.

In single inoculum, all strains formed biofilms with similar biomasses as determined by CFU/ml (Fig 6A). Biofilms co-inoculated with equal ratios of pneumocin secreting competitor to sensitive strains resulted in complete elimination of the sensitive strain which did not allow us to determine transformation efficiency presumably because the sensitive donor strains were eliminated too early to contribute to DNA exchange (S1 Fig). To prevent complete elimination of the donor pool, competitive biofilms were inoculated at a ratio of 1:10 competitor to sensitive strain. Using the lower inoculum of competitor:sensitive strain, both the pneumocin producing wild type and the pneumocin producing *blpAp*_G-90A_ strain had a competitive advantage over the co-inoculated sensitive strain (Fig 6B). The median competitive index of the wildtype strain was one and a half logs greater than that for the non-producer control. Notably, the *blpAp*_G-90A_ strain had an even greater advantage, with a nearly 9-fold greater median competitive index compared with the wildtype strain. These findings were consistent with the conclusion that competence independent induction of the *blp* locus is intact in the biofilm model. The single nucleotide change that diminishes ComE binding to the *blpA* promoter may actually enhance BlpR-mediated bacteriocin activity, resulting in greater killing in this model.

Competition within the biofilm involving the wildtype pneumocin-producing strain (BIR_164_) was associated with a 14 fold increased median transformation efficiency compared with the non-producing strain (BIR_D39_) (Fig 6C). This suggests that pneumocin production promotes release of DNA that is accessible for transformation above that seen with fratricide alone. Transformation efficiency calculations with the pneumocin producing *blpAp*_G-90A_ mutant demonstrated an efficiency that was significantly lower than was noted in the biofilm with the wildtype strain. This result demonstrated that, when the connection between competence and pneumocin production is lost, less transformation events occur during competition with the mutant (Fig 6C).

## Discussion

*Streptococcus pneumoniae* has persisted as a prominent member of the nasopharyngeal community and a cause of significant disease largely as a result of its remarkable capacity for adaptation through horizontal gene transfer. Competence also allows pneumococci to repair chromosomal damage through the uptake of homologous DNA from the environment [25,33]. Large bacterial population studies have demonstrated that the majority of DNA that is incorporated into the pneumococcal genome is derived from other pneumococci [1,34]. The immediate source of DNA for these recombination events is likely to come from neighboring bacterial cells. Most pneumococci that have been studied secrete one of two major CSP types and have intact competence systems. Presumably, in nature, neighboring pneumococcal strains that share the same CSP type are likely to communicate through the shared induction of the competence state resulting in production of fratricide immunity.

Unlike the competence mediated fratricide system, the *blp* bacteriocin locus is characterized by diversity in both the signaling peptides as well as the effector and immunity proteins [20,21,23,24]. Neighboring strains can produce one of four major BlpC types and may produce a variety of different pneumocins with their associated immunity proteins. Given this diversity in signaling and effector proteins, competence mediated induction of the *blp* locus would be predicted to provide a competitive advantage to strains beyond that provided by fratricide effectors alone. Pneumocin production by itself may be sufficient to release DNA from a wide variety of neighboring strains, however, cross-regulation of competence and pneumocin production can enhance the prey to predator exchange of genetic material by coordinating the timing of the two systems. Biofilm experiments were performed using strains that were matched in CSP type allowing us to determine the additional contribution of pneumocin production to DNA exchange compared with that mediated by fratricide effectors alone. Because we did not test pneumocin producing strains that were deficient in fratricide effector production in this model, it is unclear if the advantage in pneumocin-attributed transformation events requires the cooperation of these effectors.

An advantage of the biofilm experiments is that it has allowed us to address the impact of the substantial gap in time between competence development and BIR induction that we noted in broth grown organisms. Experiments using planktonically grown organisms showed an at least one hour delay between CSP addition and upregulation of the genes encoding pneumocins and immunity proteins. This delay is due to the time needed for the accumulation of freely diffusing BlpC to reach a critical concentration that would signal the locus. Because the competence state in pneumococcus is tightly controlled in broth culture, with transformability lasting for no more than 30–40 minutes [35], we wondered whether the delay in the production of pneumocins might occur too late to impact availability of DNA. The comparison of genetic exchange between co-inoculated biofilm grown organisms supports the role of pneumocins in the liberation of DNA from neighboring strains that is then available for transformation. The enhanced ability of pneumocin producing strains to take up DNA suggests that the delay noted in broth grown organisms is not a significant factor in the dense micro-communities found in biofilm grown organisms. This may be because DNA released from pneumocin targeted cells is sufficiently stable in the biofilm to provide a source of DNA for transformation when, perhaps, a subsequent wave of competence occurs. Alternatively, it may be that in dense micro-communities where diffusion is limited, the kinetics of pheromone accumulation is faster than in broth where the peptides can diffuse freely. The lower transformation efficiency of the *blpAp*_G-90A_ mutant strain compared with the wildtype strain in competitive biofilms suggests that the regulatory link between the two systems is important in adaptation, however, because the *blpAp*_G-90A_ mutant inhibited the growth of the donor pool to a greater degree than the wildtype strain, we cannot be certain that the decrease in transformation events in this strain is a direct result of the unlinking of the two systems or due to a decreased donor pool. In support of the impact of local population density on pheromone signaling, Yano *et al* have shown that agglutinating antibodies to the polysaccharide capsule of pneumococcus can augment the response to quorum sensing signals [36]. Consistent with our findings, they showed that *blpA* and *blpC* were induced within 8 minutes of CSP addition when administered in the presence of the agglutinating antibody. Moreover, agglutinated pneumococcus showed induction of bacteriocin and competence genes even without the addition of exogenous inducer pheromones, suggesting the importance of local bacterial density. Additionally, the duration of competence has only been determined under broth grown conditions; the regulatory mechanism that controls the competence system may function very differently in biofilm grown organisms. In support of this, Marks et al have recently shown that *comD* and *comX* transcripts can be detected at high levels in biofilm grown organisms at 24, 48 and 72 hours of incubation [31]. This suggests that either competence is constitutive in this biofilm model, or that localized waves or spots of competence are occurring throughout biofilm growth.

We have previously shown that strains carrying a frame shift mutation or deletion in the *blpA* gene do not secrete BlpC and lack inhibitory activity in plate assays [24]. Repair of the frame shift mutation in a naturally disrupted strain restored both spontaneous activation of the locus on plates and inhibitory activity in overlay assays demonstrating the importance of the BlpAB transporter in BlpC secretion and bacteriocin activity under these non-competence promoting conditions. In this study, we showed that the competence transporter, ComAB can both cleave and secrete a fraction of BlpC independently of BlpAB. The contribution of ComAB to BlpC secretion is most notable in strains that lack a functional *blpA* gene. This finding may have important implications in the nearly 70% of the *S*. *pneumoniae* population that have either a frame shifted or large deletion that disrupts the *blpA* gene. The role of ComAB secretion will be particularly important in environments where competence is likely to be induced. Experiments with the chimeric strains demonstrated that CSP can induce BlpC secretion in a ComAB sufficient background, but little to no secretion is seen when ComAB is absent, even in the presence of an intact *blpA* gene. This data suggests that ComAB may play an important role in the early secretion of BlpC after CSP stimulation. It seems likely that ComAB can augment secretion before sufficient BlpAB is made or can support BlpC secretion when BlpA is absent, either of which may allow for accumulation of BlpC in the environment. Unlike wildtype reporter strains, we suspect that the chimeric and BlpC_FLAG_ strains may produce very little BlpA under unstimulated conditions due to the absence of a functional BlpC, making the contribution of ComA more dramatic in these backgrounds. Consistent with this, we found that *comAB* deletion strains that express only BlpAB showed natural BIR induction even in competence non-permissive conditions while the *blpA*_*FS*_ strain that expresses only ComAB showed BIR induction exclusively in competence permissive media. This suggests that under conditions where competence induction is not favored, the contribution of ComAB to BlpC secretion is functionally negligible and all BlpC secretion is mediated by BlpAB.

Our studies have shown that ComAB mediated secretion of BlpC is modest compared with that mediated by BlpAB, however, ComAB mediated BlpC secretion is likely to be sufficient to stimulate BIR transcription during the development of competence. This implies that the cheater strains that were previously shown to only produce bacteriocin immunity in response to BlpC secreted by competitors may actually retain the capacity to secrete their own BlpC during the competence state. Because competence is considered a stress response, it is possible that this mechanism becomes important during pneumocin mediated killing, potentially allowing a strategy for self-protection. This hypothesis was not directly tested in our biofilm assays because, although the sensitive strain used has a disrupted *blpA* gene, it does not encode immunity proteins that would protect against the pneumocin producing strain.

Our studies did not address whether pneumocin (bacteriocin) secretion itself might also be supported by ComAB in the absence of a functional BlpA. In plate and animal studies, strains with a non-functional version of BlpA have consistently failed to show an inhibitory phenotype [24] which argues against a prominent role of ComAB in the secretion of pneumocins.

Other Streptococcal species have bacteriocin loci that are directly induced during competence [37,38]. There is speculation that, given the similarity between the *blp* and *com* systems, the two loci are the result of a remote gene duplication and subsequent specialization [39]. Martin *et al* have noted that only a few related *Streptococcal* species have both *blpRH* and *comDE* regulatory systems, while most others carry homologs of one or the other but not both systems. This has led to the suggestion that the *blp* locus was added to an ancestor of the *S*. *mitis* group to provide two distinct systems that regulate competence and bacteriocin production separately. We demonstrated that a single nucleotide difference in the BlpR binding site can support ComE mediated up-regulation of locus through increased production and secretion of BlpC. The guanine at position -90 from the *blpA* start codon is highly conserved in all pneumococcal genomes studied to date, suggesting that cross regulation between competence and pneumocin systems is likely to be universal in pneumococcus. This cross talk seems to only go in one direction, there is currently no evidence that BlpC stimulation can induce competence [20,32]. Using either competence deficient strains or the single nucleotide mutant, we have shown that pneumocin production can be independent of competence in biofilm and overlay assays suggesting that the stimulation of each regulon is not interdependent under all conditions. Interestingly, our data suggests that the single nucleotide at position -90 that supports ComE binding appears to also make the *blpABC* promoter less sensitive to BlpR stimulation. The trade-off for allowing for *com/blp* cross talk in this case is decreased responsiveness to BlpC stimulation. The separation of the two systems might be important in *Streptococcus pneumoniae* to support competitive interactions under conditions where the significant phenotypic changes induced by competence are not favored. The maintenance of cross talk between the two systems during competence induction, however, is likely to be advantageous because of the potential impact on adaptation by allowing pneumocin secreting strains access to a larger pool of DNA. The exposure to a greater diversity of genetic material may augment acquisition of new virulence factors such as altered capsule loci or new antibiotic resistance genes and therefore allow for better survival under selective host conditions.

## Supporting Information

S1 Fig Results of a competitive biofilm using a 1:1 ratio of pneumocin producer D39BIR_P164_ (spectinomycin R) to sensitive strain D39BIR_D39_ (streptomycin R). When competed at a 1:1 ratio, the bacteriocin producer strain (blue circles) completely eliminates the sensitive strain. When competed at this ratio, all streptomycin resistant strains that are recovered from the biofilm (green squares) are also spectinomycin resistant (orange triangles). Because the spectinomycin resistance marker is linked to the *blp* locus, this finding is consistent with movement of the streptomycin resistance marker into the bacteriocin producer strain and elimination of the sensitive pool.
(PDF)
