# Supplementary material for: Coordinated Bacteriocin Expression and Competence in Streptococcus pneumoniae Contributes to Genetic Adaptation through Neighbor Predation
Source: PLoS Pathog. 2016 Feb 3;12(2):e1005413. doi: 10.1371/journal.ppat.1005413 (PMC4739721; doi:10.1371/journal.ppat.1005413)
Supplement: S1 Fig — When competed at a 1:1 ratio, the bacteriocin producer strain (blue circles) completely eliminates the sensitive strain. When competed at this ratio, all streptomycin resistant strains that are recovered from the biofilm (green squares) are also spectinomycin resistant (orange triangles). Because the spectinomycin resistance marker is linked to the blp locus, this finding is consistent with movement of the streptomycin resistance marker into the bacteriocin producer strain and elimination of the sensitive pool. (PDF) [file ppat.1005413.s001.pdf]

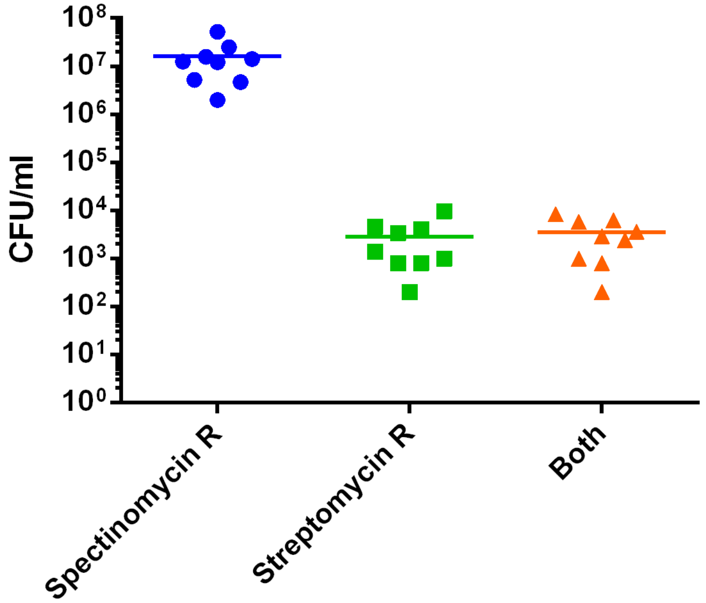

Figure S1. Results of a competitive biofilm using a 1:1 ratio of pneumocin producer D39BIR$_{P164}$ (spectinomycin R) to sensitive strain D39BIR$_{D39}$ (streptomycin R). When competed at a 1:1 ratio, the bacteriocin producer strain (blue circles) completely eliminates the sensitive strain. When competed at this ratio, all streptomycin resistant strains that are recovered from the biofilm (green squares) are also spectinomycin resistant (orange triangles). Because the spectinomycin resistance marker is linked to the *blp* locus, this finding is consistent with movement of the streptomycin resistance marker into the bacteriocin producer strain and elimination of the sensitive pool.
